# DO THE IMMUNOSUPPRESSIVE DRUGS AFFECT THE HYPOTHALAMIC NUCLEI INVOLVED IN THE REGULATION OF FOOD INTAKE? AN EXPERIMENTAL STUDY

**DOI:** 10.1590/0102-672020210002e1636

**Published:** 2022-01-31

**Authors:** Rodrigo SCHUH, Djanira Aparecida da Luz VERONEZ, Eduardo José Brommelstroet RAMOS, Flávia Dorieux Wastner CUNHA, Mattheus Lopes PEREIRA, Jeferson de Jesus ARANHA, Marcelo Alves ARANHA, Jorge Eduardo Fouto MATIAS

**Affiliations:** 1 Universidade Federal do Paraná - Departamento de Cirurgia - Curitiba - Paraná - Brasil; 2 Universidade Federal do Paraná - Departamento de Anatomia - Curitiba - Paraná - Brasil

**Keywords:** Mycophenolic acid, Hypothalamus, Obesity, Tacrolimus, Transplants, Ácido Micofenólico, Hipotálamo, Obesidade, Tacrolimo, Transplantes

## Abstract

Immunosuppressive drugs are essential for reducing the rejection risk in post-transplant patients, which is commonly associated with this procedure. However, side effects of those drugs on the hypothalamic nuclei involved in the food intake regulation, excessive weight gain, and also associated comorbidities are still unknown.

**PURPOSE:**

The purpose of this study was to analyze possible changes in the neuronal morphology and cell density in the paraventricular nuclei, lateral hypothalamic area, dorsomedial nuclei, and ventromedial and arcuate nuclei in Wistar rats submitted to immunosuppressive treatment with tacrolimus (TAC) or mycophenolate mofetil (MMF).

**METHODS:**

Adult male Wistar rats were randomly assigned to the following groups according to the oral treatment administered for 14 weeks: control, sham (placebo), TAC (1 mg/kg of weight), and MMF (30 mg/kg of weight). After treatment, the animals were sacrificed and their brains fixed for later histological staining. Subsequently, the slides were photodocumented for stereological analysis of the hypothalamic nuclei.

**RESULTS:**

All experimental groups showed a weight gain throughout the study. There was no significant difference in neuronal density/number of cells in the hypothalamic nuclei between groups. Morphological changes were not detected in the hypothalamic neurons.

**CONCLUSION:**

Treatments with immunosuppressants could not modify the morphological and cell density aspects of the hypothalamic nuclei during this supplementation period.

## RESUMO - RACIONAL

Drogas imunossupressoras são indispensáveis para pacientes pós-transplante, diminuindo, significativamente, os riscos de rejeição inerentes a este tipo de procedimento. No entanto, seus efeitos colaterais sobre os núcleos hipotalâmicos envolvidos na regulação da ingestão de alimentos e o efeito no excessivo ganho de peso e suas comorbidades associadas são desconhecidos.

**OBJETIVO:**

Analisar a ocorrência de alterações morfológicas dos núcleos paraventricular, área hipotalâmica lateral, dorsomedial, ventromedial e arqueado em ratos Wistar submetidos ao tratamento imunossupressor com Tacrolimus (TAC) ou Micofenolato Mofetil (MMF).

**MÉTODOS:**

Foram utilizados Ratos Wistar machos adultos distribuídos, randomicamente, em quatro grupos de acordo com o tratamento oral utilizado por 14 semanas: Controle; Sham (Placebo); Tacrolimus (TAC 1mg/kg peso) e Micofenolato Mofetil (MMF 30mg/kg peso). Ao final do tratamento, os animais foram eutanasiados e seus encéfalos fixados para o processamento histológico. Posteriormente, as lâminas foram fotodocumentadas para o desenvolvimento da análise estereológica dos corpos celulares dos neurônios dos núcleos hipotalâmicos, tendo como parâmetros a densidade neuronal e no número de neurônios.

**RESULTADOS:**

Todos os grupos estudados mostraram curva de ganho de peso ponderal durante todo o período de experimento. Não houve diferença significativa na densidade neuronal e no número de neurônios hipotalâmicos dos núcleos hipotalâmicos entre os grupos estudados. Não foram detectadas alterações morfológicas dos corpos celulares dos neurônios hipotalâmicos capazes de serem imputadas ao uso dos imunossupressores envolvidos no estudo.

**CONCLUSÃO:**

O tratamento dos animais experimentais com os imunossupressores não evidenciou alterações no número e densidade dos corpos celulares dos neurônios dos núcleos hipotalâmicos estudados.

## INTRODUCTION

In the past two decades, survival rates after liver transplantation have increased to 85% in 5 years and 56% after 20 years, mainly due to advances in surgical techniques, immune management, and preoperative and postoperative care^5^. However, the increased patients’ survival undergoing liver transplantation was accompanied by an increase in the prevalence of chronic diseases, generally higher than the prevalence found in the general population. In addition, the weight gain experienced by these patients stands out, generating overweight and obesity with impact on survival ^25^.

Similarly, an increased obesity in 40% of patients in the first-year post-transplant was reported and, after 3 years of surgery, about 70% of them had excess body weight^22^.

Besides, weight gain after liver transplantation may have different factors, such as positive energy balance^21^, sedentary lifestyle^13^, development of hypometabolic state^23^, and use of immunosuppressive therapies^7^.

Therefore, there is an evidence of expressive weight gain associated with post-transplant immunosuppressive therapy in humans^1^ ^,^ ^4^ ^,^ ^13^ ^,^ ^14^.

As a result, the main hypothesis is that possible hypothalamic changes can occur as a side effect of immunosuppressive therapy with tacrolimus (TAC) and mycophenolate mofetil (MMF), causing weight gain and obesity. Actually, therapeutic interventions capable of stopping or limiting the involvement of the hypothalamus may become possible new therapeutic strategies for obesity prevention in the post-transplantation immunosuppressant treatment.

Few preclinical studies are involved in investigating the mechanisms related to immunosuppression therapies and post-transplantation obesity. Thus, the neurotoxic factors of immunosuppressive drugs and their effects on the hypothalamic nuclei involved with the regulation of food intake and energy metabolism need to be studied. Regarding this, the aim of this study was to analyze possible changes in the neuronal morphology and cell density in the paraventricular (PA) nuclei, lateral hypothalamic (LH) area, dorsomedial (DM) nuclei, and ventromedial (VM) and arcuate (ARC) nuclei in Wistar rats submitted to immunosuppressive treatment with TAC or MMF.

## METHODS

### Animals and Ethical Approval

This study was approved by the Ethics Committee for the Use of Animals in experimental research at the Federal University of Paraná [UFPR; Ethics Committee on the Use of Animals (CEUA) nº752]. For this research, 24 male Wistar rats (*Rattus norvegicus albinus*, order: Rodentia, class: Mammalia), aged 70 days, weighed between 300 and 350 g, and supplied by the Animal Science Department of Biological Sciences at UFPR, were used.

### Experimental Design

The animals were housed in appropriate cages (maximum of five animals per cage) with controlled temperature (26±1°C) and light-dark cycle (12:12 h), relative humidity of 45%, and access to water and food *ad libitum*. After the acclimatization period, the rats were randomly distributed into four groups (N=6 per group), according to the proposed immunosuppressive treatment (once daily for 14 weeks):

- Control group: No medication was administrated.
- Sham group: Placebo administration - 0.9% saline solution (SS) via gavage.
- TAC group: TAC administration (1 mg/kg) diluted in 0.9% SS via gavage.
- MMF group: MMF administration (30 mg/kg) diluted in 0.9% SS via gavage.

### Drug administration and sample collection

The medications, such as TAC and MMF, were diluted in 0.9% SS immediately before their use. For standardization purposes, dilutions were always performed by following the same protocol: 9 mg of TAC diluted in 7 ml of 0.9% SS (1.28 mg/ml); 500 mg of MMF (one tablet) diluted in 30 ml of 0.9% SS (16.7 mg/ml). After dilution, the volume offered to the animals was similar between groups, calculated according to the body weight of each animal.

The animals received the treatment daily according to the group for 14 consecutive weeks. Drugs and placebo were administered via gavage. The animals were immobilized, and the orogastric cannula coupled to a 1 ml syringe was delicately introduced via oral cavity, reaching the esophagus and stomach. After checking the cannula passage to the digestive tract, the solution was gently injected, avoiding the solution reflux and animal discomfort. At the end of 14 weeks administration period, the animals were anesthetized and sedated with ketamine (100 mg/kg) and xylazine (5 mg/kg) intraperitoneally.

After anesthesia, decapitation was performed by manual guillotine followed by trepanation and brain removal, which were then fixed in Alfac’s solution (formaldehyde (37%-40%), glacial acetic acid, and 80º ethyl alcohol) for 16 h and relocated in recipients containing 70º alcohol where they remained until the time of packaging in Paraplast^®^ resin.

### Histological Slides

For microtomy and histological staining, alternate and uniform (7 μm thick) isotropic sections (N=6 per coordinate) of the brain were obtained using the Gebrauchshinweisefur Minot-Mikrotom microtome model 1212 (E. Leitz Wetzlar).

The specific areas of the rat hypothalamus were selected according to the stereotactic coordinate (bregma=−1.56 mm and bregma=−2.40 mm)^19^.

For histological analysis, Nissl staining and slide-mounted material with Entellan^®^ were used.

### Photodocumentation and Quantification

All histological slides were photodocumented at the Multi-User Laboratory of Conventional and Confocal Fluorescence Microscopy, and these images were obtained using the CoolCube 1 - metasystems camera connected to the AxioImager Z2 microscope (Carl Zeiss, Jena, Germany), equipped with Metafer 4/VSlide automated capture software (Metasystems, Altlußheim, Germany) and observed using VsViewer^®^ software (Metasystems).

Later, to select and obtain the desired location for analysis in each cut, the images obtained were magnified up to 30-fold using the VsViewer^®^ software, with appropriate magnification and precision for adjusting the masks for location accuracy and the grids of the physical dissector, intended to delimit the quantification area. For this capture, an Asus S550C Ultrabook connected to a Hewlett Packard EliteDisplay E241i video monitor was used, in which there was a distinction between the hypothalamic areas in the brain hemispheres, generating distinct images for each antimere. Besides, Photoshop CS6 Extended^®^ software (Adobe) was used to create the masks based on the images in the Stereotaxic Guide^19^, with the aim of delimiting the nucleus area for each of these images and their corresponding antimere. Subsequently, the Physical Dissector with dimensions of 200 × 200 μm was made, specific for the cellular quantification of each nucleus.

For the stereological quantification of the neurons’ cell bodies of the hypothalamic nuclei (i.e., ARC, PA, DM, VM, and LH), the physical dissector principle was used. This system consists of the application of a frame formed by a prohibited and another permitted line, delimiting an area of 40,000 μm^2^, where only the cell bodies of the neurons of the upper plane that are within the frame or touching their permitted line are counted.

The following stereological parameters of the neurons’ cell bodies of the hypothalamic nuclei were determined: numerical density (ND) and density by area (DA).

Quantification was obtained manually with the assistance of the ImageJ^®^ software, in which all neurons were selected and identified in the physical dissector, always from the quantification lines and their restrictions.

To obtain the three-dimensional parameters of the hypothalamic nuclei (i.e., ARC, PA, DM, VM, and LH), the following equations were applied:

ND of neuron cell bodies is:

$$Vol\left[dissector\right]=\;t.TA$$

$$ND=\;SQ/Vol\left[dissector\right](1/mm3)$$

where t is the thickness of the histological section, TA is the test area of the upper plane, and SQ is the number of neurons cell bodies.

To obtain the DA, the following equation is “used”:

DA=N/TA (µm3)

where N is the number of cell profiles observed in the test area.

### Statistical Analysis

For statistical analysis, the normal distribution of the data was assessed by the Kolmogorov-Smirnov test, and the data were considered parametric. To evaluate the weight of the animals, a two-way analysis of variance (ANOVA) test was performed, followed by the Bonferroni post-test. Student’s t-test was performed to assess possible differences between cerebral hemispheres in the evaluated nuclei. Differences between groups, considering the two hemispheres of the sections, were assessed by one-way ANOVA followed by the Newman-Keuls post hoc test. Values were expressed as mean ± standard error of the mean, and the level of significance was set at p=0.05.

The software used for statistical analysis and graph generation was GraphPad Prism^®^ (version 5.01).

## RESULTS

**Figure 1 -:**
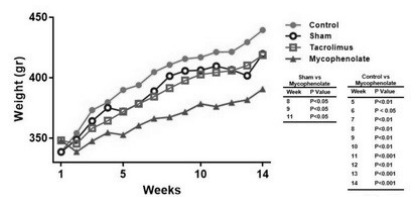
Demonstration of animal weight over 14 weeks of treatment and p=0.05 values. Two-way ANOVA. N=6 per group.

All animals gained weight throughout this study (Figure 1); however, it was not possible to identify a significantly increased weight in animals treated with TAC immunosuppressive compared to the control and sham groups. Analyzing the MMF group, there was less weight gain compared to the sham (8, 9, and 11 weeks) and control (5-14 weeks) groups.

### Paraventricular Nucleus

**Figure 2 -:**
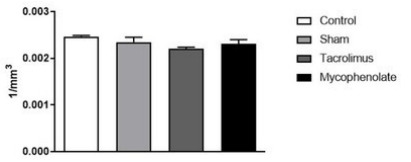
Paraventricular neurons for morpho-quantitative study. One-way ANOVA. Mean ± error of the mean. N=6 per group.

Comparing neuronal density of the PA nuclei between groups (Figure 2), it was observed that there was no statistical difference between them [F (3.20)=2.327; p=0.1054].

### Lateral Hypothalamic Area

**Figure 3 -:**
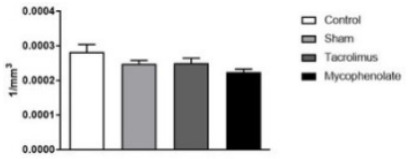
Lateral hypothalamic area neurons for morpho-quantitative study. One-way ANOVA. Mean ± error of the mean. N=6 per group.

The neuronal density of the LH area did not show significant variations (Figure 3) when compared between the groups studied [F (3.20)=2.618; p=0.0792].

### Dorsomedial Nucleus

**Figure 4 -:**
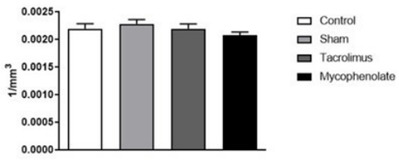
Dorsomedial neurons for morpho-quantitative study. One-way ANOVA. Mean ± error of the mean. N=6 per group.

The area of DM nuclei presented similar values (Figure 4), concerning the neuronal density analyzed in each study group [F (3.20)=1.104; p=0.3707].

### Ventromedial Nucleus

**Figure 5 -:**
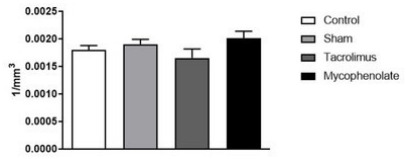
Ventromedial neurons for morpho-quantitative study. One-way ANOVA. Mean ± error of the mean. N=6 per group.

Considering the area of the VM nuclei, no statistical difference was detected between the groups (Figure 5) when neuronal density was calculated [F (3.20)=1.641; p=0.2117].

### Arcuate Nucleus

**Figure 6 -:**
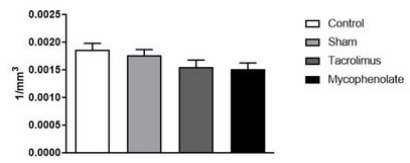
Arcuate neurons for morpho-quantitative study. One-way ANOVA. Mean ± error of the mean. N=6 per group.

The area of ARC nuclei also didn’t show a significant difference (Figure 6) between the neuronal density measured for each study group [F (3.20)=2.133; p=0.1281].

## DISCUSSION

The experimental model was developed in line with the general principles of the Brazilian Guidelines of Care and Animals Use for scientific and educational purposes^15^, which encourages the adherence and application of the replace, reduce, and refine (3Rs) principles. The animals treated with TAC and MMF for the experimental model of the quantitative analysis of neurons were obtained from the reuse of male albino Wistar rats (*R. norvegicus albinus*, order: Rodentia, class: Mammalia) from previous research: “Assessment of spermatogenesis in immunosuppressive rats,” under the protocol nº752, certified by the CEUA, Biological Sciences Sector of the UFPR. Thus, the animals’ experimental use was made for the development of this assay to the quantification of neurons in the PA, LH, DM, VM, and ARC hypothalamic nuclei in male Wistar rats treated or not with the immunosuppressants such as MMF and TAC. Accordingly, the control group was formed, without medication administration; the TAC group was formed with the administration of a daily dose of TAC (1 mg/kg weight) diluted in 0.9% SS; the MMF group was formed with the administration of a daily dose of MMF (30 mg/kg weight) diluted in 0.9% SS, and the sham group was formed with the administration of 0.9% SS. The gavage method was used for drug/vehicle administration.

The stereological method for the morpho-quantitative study of the cell bodies in the hypothalamic nucleus was defined as an efficient method in estimating neuronal density and the total number of hypothalamic neurons for later comparison between untreated Wistar rats and those treated with immunosuppressants such as TAC and MMF. However, due to the anisotropic neuronal distribution of the hypothalamus, it was not possible to obtain the volumetric density of each hypothalamic nucleus associated with the regulation of food intake.

With the preclinical protocol used, in an attempt to mimic what happens to post-transplanted humans, no quantitative (neuronal density) or qualitative (cell morphology) differences were found in the hypothalamic nuclei studied. Surprisingly, no similar research protocol was found in the literature to analyze the effects of this immunosuppressive therapy (TAC and MMF) on the hypothalamus of Wistar rats and its relationship with obesity. Nevertheless, some authors have investigated the changes caused by obesity in the monosodium glutamate-injected models due to cellular neurotoxicity in the hypothalamus^9^ ^,^ ^11^.

Furthermore, there are only few reports about the immunosuppressant neurotoxic character in the hypothalamus. Thus, the approach regarding TAC and MMF as etiological factors related to obesity is minimal^27^. The direct correlations between immunosuppressants, hypothalamic nuclei, and obesity are still unclear, as described in the literature^1^, referring to the use of immunosuppressive drugs as a risk factor for excess weight post-transplant which is still controversial.

Conversely, several studies report significant weight gain in post-transplant patients. This weight gain can often be excessive, resulting in obesity accompanied by its associated comorbidities. That situation is a possibly growing public health problem related to patients undergoing kidney, liver, and heart transplants^1^ ^,^ ^14^.

No marked weight gain was identified in the independent groups treated with TAC and MMF. Therefore, it was not possible to verify a significant increase in weight in the animals treated with MMF when compared to the control and sham groups. Probably, this variation in weight gain is explained primarily to the absence of an associated drug group (TAC + MMF) and secondarily to the short treatment period.

In a hindlimb transplantation model, there was no weight gain with the associated use of TAC with triptolide, intraperitoneally, for 150 days in experimentally transplanted animals^10^. This can be explained by the triptolide inhibition of weight gain induced by TAC. However, this treatment protocol was conducted for a prolonged period, compared to this study, with a difference of 52 days, which could be crucial, considering the lifetime of the Wistar rat.

Likewise, several studies have shown that the drug combined interaction may be responsible for the weight gain of post-transplant patients, which commonly occurs in clinical practice^12^ ^,^ ^17^ ^,^ ^18^ ^,^ ^22^. Some authors reported excessive weight gain, including obesity in 15% of patients after liver transplants, treated with the association of immunosuppressive drugs, TAC and cyclosporine^4^ ^,^ ^6^. In contrast, with TAC and cyclosporine administrated individually, no significant weight gain was observed^6^. Similarly, transplanted individuals treated with cyclosporine at the end of the first year acquired a higher risk of obesity than those treated with TAC in the same period^8^. Therefore, some studies corroborate the absence of significant weight gain in animals treated only with TAC.

The stereological method used for the morpho-quantitative study of the neurons’ cell bodies of the hypothalamic nuclei proved to be efficient for the acquisition of neuronal density and the total number of hypothalamic neurons for later comparison between untreated and treated Wistar rats with immunosuppressants, TAC and MMF. However, due to the anisotropic neuronal distribution of the hypothalamus, it was not possible to obtain the volumetric estimate of each hypothalamic nucleus involved with the regulation of food intake.

In this study, the treatment with TAC and MMF immunosuppressants was not responsible for a significant change in neuronal density in the hypothalamic nuclei, such as PA, LH, DM, VM, and ARC. That data can be explained, possibly, by the absence of a specific treated group, simultaneously with TAC and MMF as conventionally occurs in immunosuppressive therapies of post-transplant patients at the clinic.

The absence of morphoquantitative changes in hypothalamic neurons in the different study groups does not confirm the absence of immunosuppressant effects. Thus, complementary methods are needed to assess the neurotoxic effects of immunosuppressants on those brain regions. According to the literature data^22^, there is a possible loss of afferent and efferent pathways between the liver and the hypothalamus during liver transplant surgery, leading to a disturbance of the liver’s role in metabolic homeostasis, which can delay postprandial satiety and, therefore, can directly influence excessive food consumption. However, no conclusive data have been established to determine whether this disorder has a direct effect on food intake and body mass in post-transplant patients. These data point to different changes from those investigated in this study, indicating that alterations in hypothalamic neuronal density and morphology may not be directly related to the weight gain found in patients. In addition, the animals at the present study were not subjected to any surgical intervention similar to the liver transplantation procedure; therefore, further studies are needed to investigate the neuronal density and morphology in a post-transplant animal model.

There is no consensus on studies that investigate the effects of immunosuppressants on the hypothalamus, more specifically in the hypothalamic nuclei studied, regarding their role in cytoarchitecture in this area. However, in the literature, possible changes in hypothalamic nuclei were cited to justify the disorganization of hunger and satiety control exerted by hypothalamic neurons involved in the development of obesity^3^ ^,^ ^20^.

Furthermore, based on experimental models, through the injection of high concentrations of monosodium glutamate in mice, the rapid gain of animal weight was reported due to the high toxicity caused in the cells of the hypothalamic nuclei involved with food intake^24^ ^,^ ^28^.

The package leaflet approved by the National Health Surveillance Agency (ANVISA) on October 29, 2015, of the drug Cellcept^®^, used in the study as a treatment with MMF, informs that in the tests performed, between 3% and 10% of patients had vertigo, depression, seizures, tremors, neuropathies, hallucinations, delirium, among other neurological effects and, in addition to these, the presented weight gain was classified as very common in cases of heart transplants and common when these patients were submitted to kidney and liver transplants. However, it was also reported that MMF has no neurotoxic effect^2^ ^,^ ^16^, similar to the MMF group in the present study.

Furthermore, due to the principles of the 3Rs^15^, there were some limitations as to the specificity in causing neurotoxicity and direct impairment of the hypothalamic nuclei related to the control of food intake and, therefore, triggering obesity. However, the sequential use of animals in this study does not invalidate the results obtained in the experimental model developed with animals treated with TAC and MMF.

Finally, the drug interaction between immunosuppressants and other drugs could be responsible for the neurotoxic condition of certain regions of the nervous system, thus favoring weight gain and the development of obesity; however, future preclinical studies are needed to confirm this hypothesis.

## CONCLUSIONS

Treatments with isolated immunosuppressants, such as TAC and MMF, could not modify the morphological and cell density aspects of the hypothalamic nuclei during this supplementation period. In addition, no obesity was observed with the immunosuppressant administration protocol used.
